# *Mimulus peregrinus* (Phrymaceae): A new British allopolyploid species

**DOI:** 10.3897/phytokeys.14.3305

**Published:** 2012-07-06

**Authors:** Mario Vallejo-Marín

**Affiliations:** 1 Biological and Environmental Sciences, University of Stirling. Stirling, Scotland. FK9 4LA

**Keywords:** Allopolyploidy, *Erythranthe*, hybrid evolution, introduced species, *Mimulus guttatus*, *Mimulus luteus*, rapid evolution, speciation

## Abstract

Polyploidization plays an important role in species formation as chromosome doubling results in strong reproductive isolation between derivative and parental taxa. In this note I describe a new species, *Mimulus peregrinus* (Phrymaceae), which represents the first recorded instance of a new British polyploid species of *Mimulus* (2n = 6x = 92) that has arisen since the introduction of this genus into the United Kingdom in the 1800’s. *Mimulus peregrinus* presents floral and vegetative characteristics intermediate between *Mimulus guttatus* and *Mimulus luteus*, but can be distinguished from all naturalized British *Mimulus* species and hybrids based on a combination of reproductive and vegetative traits. *Mimulus peregrinus* displays high pollen and seed fertility as well as traits usually associated with genome doubling such as increased pollen and stomata size. The intermediate characteristics of *Mimulus peregrinus* between *Mimulus guttatus* (2n = 2x = 28)and *Mimulus luteus* (2n = 4x = 60-62), and its close affinity with the highly sterile, triploid (2n = 3x = 44-45) hybrid taxon *Mimulus × robertsii* (*Mimulus guttatus* × *Mimulus luteus*), suggests that *Mimulus peregrinus* mayconstitute an example of recent allopolyploid speciation.

## Introduction

The genus *Mimulus* (Phrymaceae) comprises more than 120 species, the majority (75%) of which occur in western North America, and the remaining having a world-wide distribution including Eastern North America, South America, Australia, the Himalayas, Japan and Madagascar (Grant 1924, Beardsley and Olmstead 2002, Wu et al. 2007). Species of *Mimulus* have been spread outside their native range due to deliberate and accidental introductions. For example, *Mimulus guttatus*, a native of western North America, is now found in New Zealand and more than 16 European countries (Truscott et al. 2008, van Kleunen and Fischer 2008, Tokarska-Guzik and Dajdok 2010). In some of these areas of introduction, *Mimulus guttatus* has become naturalized and widely distributed, forming a nontrivial component of the local flora (e.g. Wales, Northern England, Scotland, Poland, Germany and New Zealand; Roberts 1964, Stace 2010, Tokarska-Guzik and Dajdok 2010, Vallejo-Marin unpublished). In the United Kingdom (UK), naturalized populations of *Mimulus* are widespread (Preston et al. 2002), and the genus is represented here by three currently extant species (*Mimulus guttatus*, *Mimulus luteus* and *Mimulus moschatus*), and a complex array of interspecific hybrids, some of which are locally invasive (Stace 2010).

One of the most conspicuous hybridization complexes in the UK involves closely related taxa, of isolated geographic origin: the North American *Mimulus guttatus* DC. (2n = 28, 30, 56, with most North American and British plants 2n = 2x = 28, Mukherjee and Vickery 1962, Vickery 1995, Stace 2010), and the South American taxa *Mimulus luteus* L. (2n = 4x = 60, 61, 62) and *Mimulus cupreus* Dombrain (2n = 4x = 62) (Stace 2010). These taxa belong to *Mimulus* Section *Simiolus* Greene (= *Erythranthe* Section *Simiola* (Green) Nesom & Fraga; Barker et al. 2012). Crosses between *Mimulus guttatus* and *Mimulus luteus*/*Mimulus cupreus* yield sexually sterile triploids (2n = 3x = 44, 45, 46) that are nevertheless vegetatively vigorous (Roberts 1964, 1968, Stace 2010). In the UK, hybrids between *Mimulus guttatus* and *Mimulus luteus*/*Mimulus cupreus* have been grown since the 1800’s and some of them have become well established throughout the country. For instance, the hybrid between *Mimulus guttatus* and *Mimulus luteus* (= *Mimulus × robertsii*; Silverside 1990) escaped cultivation at least by 1872 (Preston et al. 2002), and currently forms numerous naturalized populations with a scattered distribution in the British Isles (Preston et al. 2002, Stace 2010, BSBI 2011).

Despite being widely distributed and having persisted in the UK for 140 years, the evolutionary fate of *Mimulus guttatus* × *Mimulus luteus*/*Mimulus cupreus* triploid hybrids has been thwarted by their high pollen- and seed-sterility (Mukherjee and Vickery 1962, Roberts 1964, 1968, McArthur 1974, Parker 1975). Sterility is common in hybrids produced by the merging of genetically differentiated genomes (Mallet 2007), including cases when parents have different chromosome numbers (Stebbins 1950, Stebbins 1958). When hybridization gives rise to viable triploids, these tend to generate high proportions of unbalanced, aneuploid, and usually non-functional gametes (Ramsey and Schemske 1998). However, sterile plant hybrids often recover fertility after genome duplication (Stebbins 1958). Polyploidization in interspecific hybrids — allopolyploidization — has been linked to the restoration of sexual fertility in some natural triploid hybrids (e.g., *Senecio*, Abbott and Lowe 2004).

Polyploidization plays a particularly important role in species formation, as chromosome doubling results in immediate and strong reproductive isolation between the derivative and parental species (Rieseberg and Willis 2007, Köhler et al. 2010). It is therefore not surprising that polyploidization is often thought to be fundamental to angiosperm diversification (Stebbins 1950, Grant 1971, Ramsey and Schemske 2002, Soltis 2005, Wood et al. 2009). In *Mimulus*, speciation by hybridization and polyploidization may have played an important role during the diversification of this group (Vickery 1995, Beardsley et al. 2004). For instance, allopolyploidization between diploid *Mimulus guttatus* and *Mimulus nasutus* has given rise to a widespread North American tetraploid taxon that is strongly reproductively isolated from its progenitors (Sweigart et al. 2008). Despite the importance of hybridization and polyploidization for plants in general, the opportunity to study early events in speciation via this route is limited by the small number of angiosperm species known to have originated via allopolyploidization in the last 150 years (e.g. *Spartina anglica* (Ayres and Strong 2001),* Tragopogon mirus, T. miscellus* (Soltis et al. 2004, 2012), *Senecio cambrensis* and *Senecio eboracensis* (Abbott and Lowe 2004)). The discovery of a recently formed polyploid hybrid species in the wild therefore would provide a window of opportunity to study the evolution and speciation of polyploid taxa.

In this note, I describe a new, fertile, polyploid (2n = 6x = 92) species of *Mimulus* (Phrymaceae), *Mimulus peregrinus*, which has currently been found in a single locality in the Lowther Hills, Scotland. A comparison of vegetative and reproductive morphology, DNA content, and chromosome number of this new polyploid species against other British *Mimulus*,strongly suggests a hybrid origin for *Mimulus peregrinus* and a close affinity with the sterile triploid hybrid *Mimulus × robertsii*. I speculate that *Mimulus peregrinus* may represent the hexaploid derivative of a hybrid between *Mimulus guttatus* and *Mimulus luteus*, although a careful examination of additional populations of both parental and hybrid taxa is required to elucidate the genetic origin, extent and distribution of this new polyploid species. If an allopolyploid origin is demonstrated, *Mimulus peregrinus* has the potential to serve as a study system to understand the evolutionary processes associated with the origin of species through hybridization and polyploidization following the breakdown of geographic barriers caused by human-assisted dispersal.

## Methods

Field surveys in August 2011 uncovered the existence of fertile individuals in a large population of *Mimulus × robertsii* in South Lanarkshire, Scotland. To further investigate these unusual plants, open-pollinated seeds were collected on 27 August 2011 from multiple seed-bearing fruits in a single patch at Shortcleuch Waters, near Leadhills, South Lanarkshire, Scotland (NS 9029 1578; 55.4237°N, 3.7349°W). Field-collected seeds—accession number 11-LED-seed—were germinated and grown in a controlled environment cabinet (Microclima 1750E; Snijders Scientific, Tilburg, the Netherlands) at the University of Stirling under 16 light-hours at 24°C and 8 dark-hours at 16°C, and 70% constant humidity. Individual plants were grown in 0.37 l round pots, filled with general purpose peat-sand compost (Sinclair, Lincoln, Lincolnshire, UK), and kept on plastic trays with abundant water. Plants were sporadically treated with SB Plant Invigorator (Fargro Ltd, Littlehampton, West Sussex, UK) to control for fungal infections. Seven plants were brought to flowering (F_1_ generation; 11-LED-seed-1 to 11-LED-seed-7), and each individual plant was used to generate F_2_ offspring via manual self-fertilization of emasculated flowers kept inside the pollinator-free growth cabinet. A representative individual of this F_2_ generation (11-LED-seed-2-14) was chosen as the holotype for the type description presented here (deposited at the Herbarium of the Royal Botanic Garden Edinburgh; E).

Pollen measurements were conducted using fresh pollen fixed in 1 ml of 70% ethanol and dyed with 50 μl of lactophenol-aniline blue (Kearns and Inouye 1993). Darkly stained grains were considered viable (Sweigart et al. 2006). Pollen diameter was measured at the widest point in expanded pollen grains using image analysis software (analySIS, Olympus Soft Imaging Solutions, Münster, Germany) at 200*×* magnification in an Olympus BX50 light microscope.

Stomata size was measured in casts obtained from the adaxiall side of healthy leaves. A negative cast was first obtained with polysiloxane precision impression material (Xantoprene VL Plus, Heraeus Kulzer Gmbh, Hanau, Germany), and a positive cast was then generated with quick-drying nail polish. Measurements of stomata length and width were done using a light microscope at 400*×*.

Chromosome counts were conducted by John Bailey (University of Leicester) in mitotic cells from root tips of two F_2_ individuals (11-LED-seed-3-21 and 11-LED-seed-5-8). Genome size was measured using DAPI-stained nuclei analysed in a CyFlow ML flowcytometer (Partec GmbH, Münster, Germany) in a commercial facility (Plant Cytometry Services, Schijndel, The Netherlands) in six F_1_ individuals (11-LED-seed-1 to 11-LED-seed-4, 11-LED-seed-6, 11-LED-seed-7). *Vinca major* was used as internal standard (2n = 92, 2C = 3.80 pg; Obermayer and Greihulber 2006). Because DAPI preferentially binds to AT-rich regions, the flow cytometry results presented here must not be treated as absolute measurements of DNA content.

## Data resources

The data underpinning the analysis reported in this paper are deposited at GBIF, the Global Biodiversity Information Facility, http://ipt.pensoft.net/ipt/resource.do?r=mimulus_peregrinus

## Taxonomic treatment

### Mimulus peregrinus

Vallejo-Marín sp. nov.

urn:lsid:ipni.org:names:77120497-1

http://species-id.net/wiki/Mimulus_peregrinus

Figure 1

Mimulus Section *Simiolus* Green (= *Erythranthe* Section *Simiola* (Green) Nesom & Fraga)

#### Type.

**United Kingdom.** Scotland: Grown from seed collected in South Lanarkshire near Leadhills, on the banks of Shortcleuch Water. Vice county 77, Ordinance Survey grid reference: NS 9029 1578. WGS84 coordinates: 55.4237°N, 3.7349°W; altitude: 360 m. 27 Aug 2011. M.Vallejo-Marín 11-LED-seed; vouchered as M.Vallejo-Marín 11-LED-seed-2-14 (holotype: E; isotypes: BM, K).

Species nova *Mimulus × robertsii* Silverside similis. Herba perennis, pollen et semen fertile, corollae, flavae, lobo centrali cum macula parva rubro. Folia ovata ad oblonga, dentata, regulariter ad irregulariter triangulo-dentata. Calyx interne cum capillis simplicibus instructis.

#### Description.

Perennial herb 5-30 cm (–1 m) high, freely rooting at the nodes. Stem erect or prostrate, glabrous below and glandular pubescent above. Leaves variable, mostly ovate-oblong 3–14 *×* 1.5–4 cm, with regular to irregularly dentate margins; basal leaves oval to spatulate, with petioles up to three-quarters as long as the blades; upper leaves ovate with much shorter petioles or sessile. Inflorescence racemose, many-flowered; pedicels 2.5–5 cm long, normally equalling or slightly longer than the corolla, but shorter in later flowers. Calyx 1.5–2.5 cm long, with 5 triangular teeth, the upper tooth distinctly longer; pubescent outside covered with glandular hairs throughout, and with short, simple hairs in the base of the calyx extending along the ridges; calyx becoming inflated in fruit, with the lower two calyx-teeth curving upwards and enclosing the fruit. Corolla ovate in frontal view, 4–5 cm wide, 3–5 cm tall, and 4–5 cm long (deep); the lobes almost truncate, particularly the two lateral ones; yellow, with a single faint-red, vertically-elongated 2 × 5 mm spot located approximately half-way on the central lower lobe; throat hairy, spotted with red, more or less open; lobes subequal, the central lower lobe slightly longer (Fig. 2). Style glabrous, ending in a bi-lobed, thigmotropic stigma. Fruit a broadly oblong capsule; seeds striate, very small (<0.02 mg; ~0.1 mm^2^). Anthers yielding abundant quantities of viable pollen (percent of viable pollen: 86.39 ± 4.01%, range: 73.24 – 96.40%, N = 6 individuals); pollen diameter from 53.43 ± 1.22 μm (mean ± SE; N =5 individuals, 100 pollen grains per individual; Hoyer’s medium) to 48.78 ± 0.97μm (mean ± SE; N =6 individuals, 100 pollen grains per individual; 70% ethanol) depending on mounting medium. Sets abundant seed following artificial self-pollination. Germination rates of self-fertilized seed 80% ± 4.2% (N =6 families, 50 seeds per family). Stomata length 35.44 μm ± 0.99 (N = 7 individuals, 20 stomata per individual). Chromosome number 2n = 92 (J. Bailey).

**Figure 1.**
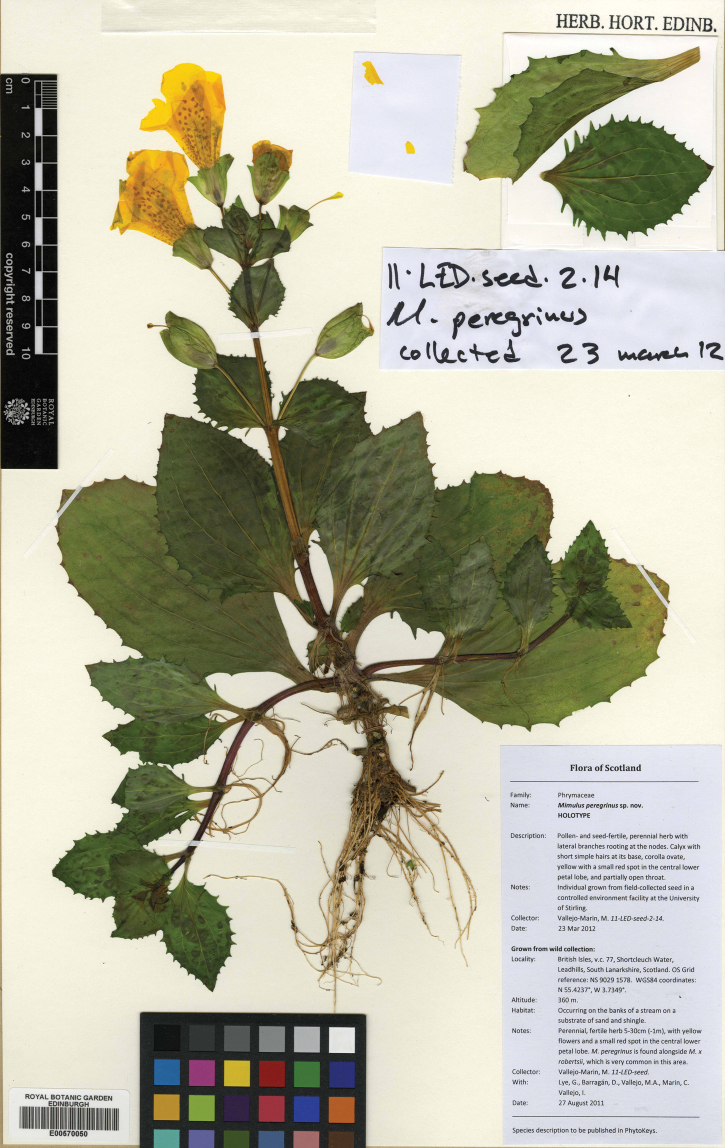
Holotype of *Mimulus peregrinus* Vallejo-Marin [11-LED-seed-2-14; barcode E00570050].

#### Distribution.

Currently known only from the banks of Shortcleuch Waters, Leadhills, South Lanarkshire, Scotland, UK (v.c. 77).

#### Ecology.

Occurring on the banks of a stream on a substrate of sand and shingle. *Mimulus peregrinus* is found alongside *Mimulus × robertsii*, whichis locally common. Flowering of *Mimulus* in this region starts in early June. Seeds of *Mimulus peregrinus* were collected in August.

#### Etymology.

The name is taken from the Latin *peregrinus* – foreigner, traveller.

#### Preliminary conservation status.

Currently known only from a single collection outside of a protected area, *Mimulus peregrinus* is provisionally assessed as Critically Endangered (CR D; population size estimated to number less than 50 mature individuals) (IUCN 2011).

#### Specimens examined.

**United Kingdom.** Scotland: Grown from seed collected at South Lanarkshire near Leadhills, on the banks of Shortcleuch Water. 55.4237°N, 3.7349°W; altitude: 360m. 27 Aug 2011. M.Vallejo-Marín, seed voucher: 11-LED-seed. All *Mimulus peregrinus* specimens examined here were derived from open-pollinated seed collected at the type locality and grown in a controlled environment. Some of these first generation seed-grown individuals (11-LED-seed-1 to 11-LED-seed-7) were then used produce a second generation via self-fertilization (e.g. 11-LED-seed-2-14).

## Discussion

**Figure 2.**
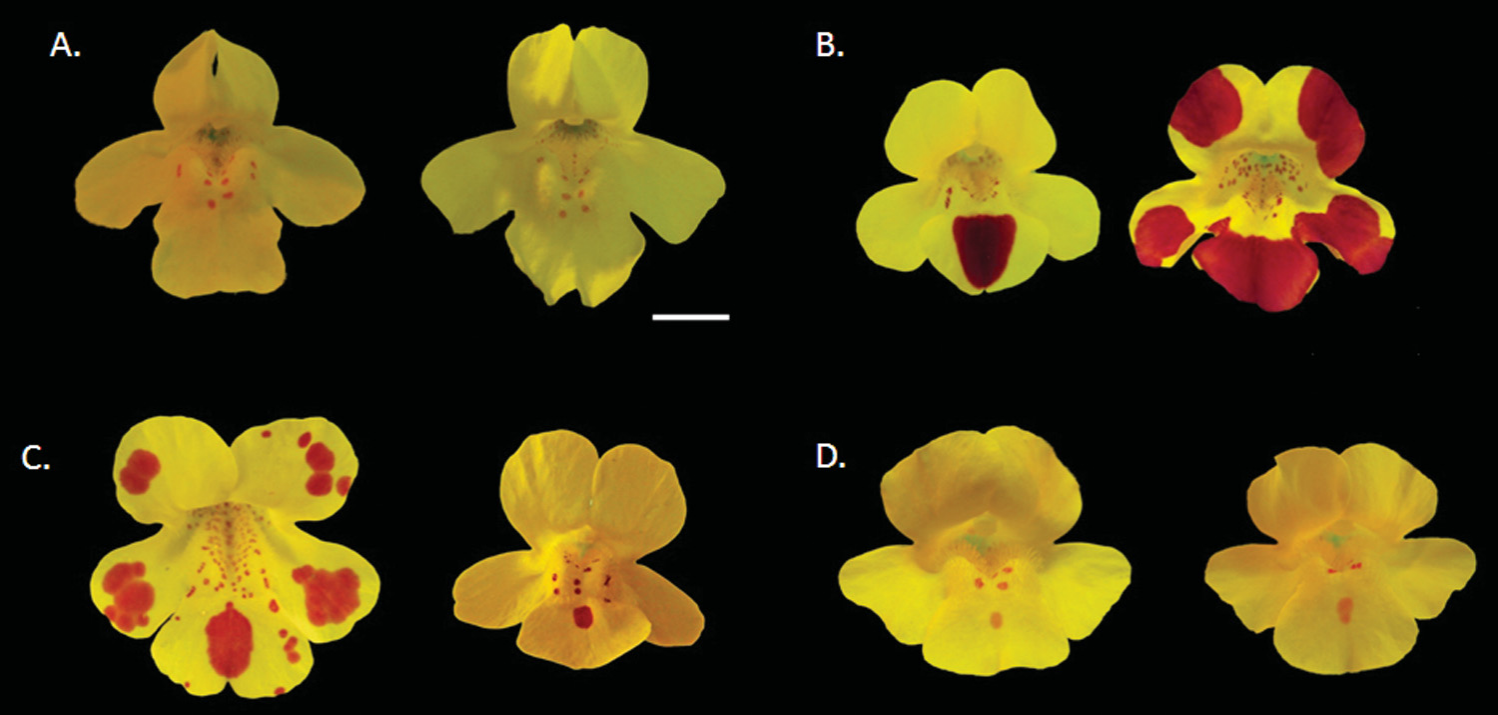
Flowers of *Mimulus peregrinus* and closely related taxa. **A** *Mimulus guttatus* **B** *Mimulus × smithii* (*Mimulus luteus luteus* × *Mimulus luteus variegatus*) **C** *Mimulus × robertsii* (*Mimulus guttatus* × *Mimulus luteus*), and **D** *Mimulus peregrinus*. Each taxon is represented by flowers from two individuals from a single locality to illustrate within-population variability: *Mimulus guttatus* = Dunblane, Perthshire; *Mimulus × smithii* = Coldstream, Scottish Borders; *Mimulus × robertsii* = Nenthall, Cumbria; *Mimulus peregrinus* = Leadhills, South Lanarkshire. Scale bar = 1cm.

*Mimulus peregrinus* can be distinguished from closely related *Mimulus* species and their hybrids in the UK based on a number of morphological and functional characters (Table 1, Fig 2). Its chromosome number, DNA content, larger stomata and pollen grain size, clearly indicate that *Mimulus peregrinus* is a polyploid species. Although the parentage of this new polyploid has not been firmly established yet, its close affinity with *Mimulus × robertsii* suggest that *Mimulus peregrinus* has been derived from hybridization between *Mimulus guttatus* and *Mimulus luteus* and thus it might have arisen through a recent (<140 years) allopolyploidization event. Below I contrast *Mimulus peregrinus* with related *Mimulus* taxa in the UK, and end with a brief discussion on its putative origin.

**Table 1.** List of main diagnostic characters differentiating *Mimulus peregrinus* from other closely related taxa of *Mimulus* found in the UK. In the cases of the very variable species *Mimulus guttatus* and *Mimulus luteus*,diagnostic characters are taken from those of British populations. For example, although *Mimulus luteus* is polymorphic for corolla-lobe red markings in Chile, the un-marked variety is not naturalized here (Stace 2010). Data presented as mean ± SE (number of individuals analyzed). Data from Stace (2010), Grant (1924) and MVM unpublished results.

**Character**	***Mimulus peregrinus***	***Mimulus guttatus***	***Mimulus luteus***	***Mimulus × smithii***	***Mimulus × robertsii***
Corolla lobes with reddish spots or blotches	Yes (one small spot in lower, central lobe)	No	Yes (a single blotch in central lower petal)	Yes (present in 1-5 lobes)	Yes (variable)
Throat of corolla	± open	± closed	± open	± open	± open
Small, simple (non-glandular) hairs on inflorescence and calyx keels	Yes	Yes	No	No	Yes
Seed fertile	Yes	Yes	Yes	Yes	No
Seed size (area in mm^2^)	0.167 ± 0.012 (6)	0.126 ± 0.008 (12)	0.103 (1)	0.112 ± 0.006 (8)	---
Seed germination	0.80 ± 0.04 (6)	0.85 ± 0.02 (11)	NA	0.47 ± 0.06 (8)	--
Pollen fertile (proportion viable)	Yes 0.864 ± 0.040 (6)	Yes 0.865 ± 0.053 (6)	Yes (NA)	Yes 0.963 ± 0.006 (2)	No 0.001 ± 0.001 (9)
Mean pollen diameter (μm) ^1^	48.77 ± 0.97 (6)	36.72 ± 0.38 (24)	44.08 ± 3.11^2^ (2)	45.09 ± 0.39 (25)	37.02 ± 1.70^3^ (9)
Stomata size (length, μm)^4^	35.44 ± 0.99 (7)	28.25 ± 0.42 (1)	NA	29.67 ± 0.55 (1)	26.83 ± 0.77 (1)
Chromosomes (ploidy)	2n = 92 (6x)	2n = 28 (2x)	2n = 59,60, 61, 62 (4x)	2n = 60, 61, 62 (4x)	2n = 44, 45 (3x); 2n = 54

^1^ = Measured in pollen preserved in 70% ethanol and dyed with lactophenol-aniline blue. ^2^ = Measured in pollen preserved in Hoyer’s medium and dyed with lactophenol-aniline blue. ^3^ = Inviable (empty) pollen grains are variable in size as they may be fully expanded or partly collapsed. ^4^ = Measured in 20 stomata per individual.

### Comparison with related *Mimulus* in Britain

**Figure 3.**
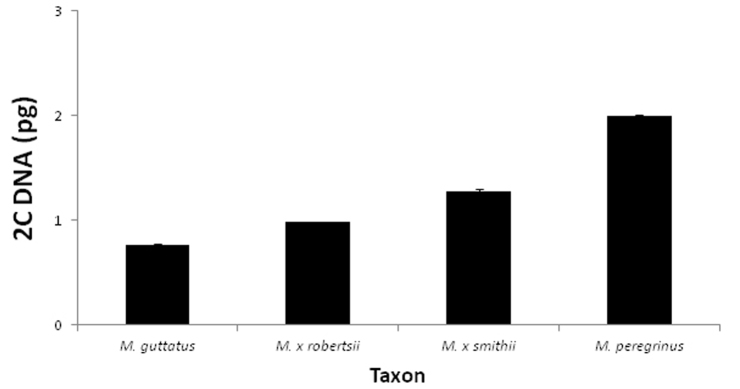
Flow-cytometry estimates of 2C DNA content (DAPI-stained) of British *Mimulus*. Error bars represent standard errors when multiple individuals per taxon were tested. Sample sizes as follows (chromosome numbers for each population are given in parenthesis when available). *Mimulus guttatus*: N *=* 4 individuals from Dunblane, Perthshire (2n = 28); and 2 individuals from Muckle Roe, Shetland; *Mimulus × robertsii* (= *Mimulus guttatus* × *Mimulus luteus*): N= 1 individual from Nenthall, Cumbria (2n = 44, 45); *Mimulus × smithii* (= *Mimulus luteus* var. *luteus* × *Mimulus luteus* var. *variegatus*): N = 2 individuals from Coldstream, Scottish Borders (2n = 59, 60, 61, 62); *Mimulus peregrinus*: N *=* 6 individuals from Leadhills, South Lanarkshire (2n = 92).All chromosome counts kindly provided by J. Bailey.

1. *Mimulus guttatus* DC. (Section *Simiolus* Green) (yellow monkeyflower). *Mimulus peregrinus* has a more open corolla throat, in contrast to the nearly closed corolla throat of *Mimulus guttatus*. The 2–5 mm red spot in the central lower lip of *Mimulus peregrinus*, is absent in most British populations of *Mimulus guttatus*. The margins of the lower leaves of *Mimulus peregrinus* are more triangular and regular than those of most *Mimulus guttatus*, although leaf traits are highly variable in the genus. Field and herbarium specimens could potentially be distinguished by the much larger size of the pollen grains in *Mimulus peregrinus*. Chromosome number and genome content as measured in flow cytometry are also diagnostic characters to distinguish these two species (Table 1, Fig. 3).

2. *Mimulus luteus* L. (Section *Simiolus* Green) (blood-drop emlets). *Mimulus luteus*, is a group of polymorphic perennial herbs comprising several interfertile varieties that are distinguished based on the presence, size and colour of markings on the corolla lobes. Taxa in this group include *Mimulus luteus* var. *rivularis* Lindl. 1826, with a single large red spot on the middle lower lip; *Mimulus luteus* var. *variegatus* (Lodd.) Hook 1834, with pale yellow corollas tinted with pink at the lobe margins; and *Mimulus luteus* var. *youngana* Hook 1834 (= *Mimulus smithii* Lindl 1835, not Paxton), with deep yellow corollas and lobes with large red spots at the margins (Grant 1924). In the UK, many extant populations of *Mimulus luteus* likely represent crosses between taxa in this interfertile group (e.g. *Mimulus luteus* var. *rivularis* × *Mimulus luteus* var. *variegatus*) (MVM pers. obs.), and present highly variable patterns of spots and blotches in the corolla lobes. *Mimulus peregrinus* can be distinguished from most species and hybrids in the *Mimulus luteus* aggregate by its more robust habit, elliptical leaves with dentate and slightly irregular margins, and the presence of only a small, faint, elongated central spot in the lower lip. Most importantly, *Mimulus peregrinus* possesses simple hairs in the calyx, which are always absent from all varieties of *Mimulus luteus*. Other diagnostic characters of *Mimulus peregrinus* are pollen grain size, stomata size, DNA content as measured in flow cytometry and chromosome number (Table 1, Fig. 3).

3. *Mimulus cupreus* Dombrain (Section *Simiolus* Green) (copper monkeyflower). *Mimulus cupreus* with orange to yellow corollas, and which is closely related to *Mimulus luteus*, has been reported in the UK but most likely in error for the hybrid between *Mimulus guttatus* and *Mimulus cupreus* (*Mimulus × burnetii* S. Arn.) (Stace 2010). In contrast with *Mimulus peregrinus*, the copper monkeyflower usually has orange corollas, more open corolla throat, lacks simple hairs in the calyx, and has a smaller chromosome complement (2n = 62).

(3) *Mimulus moschatus* Douglas ex Lindl. (Section *Paradanthus* Grant) (musk). *Mimulus moschatus* is easily distinguished from other British *Mimulus* including *Mimulus peregrinus* by its smaller yellow corollas (1–2.5 cm), glandular-hairy pubescence throughout the plant, and chromosome number (2n = 4x = 32 × = 8, 9, 10, Vickery 1995). *Mimulus moschatus* does not hybridize with other British *Mimulus*.

(4) *Mimulus × robertsii* Silverside (*Mimulus guttatus* × *Mimulus luteus*). A highly pollen- and seed-sterile, perennial herb rooting at the nodes, its yellow flowers are marked with orange to red to brown spots of various sizes in the petal lobes (Roberts 1964, Silverside 1990, Silverside 1998,Stace 2010). The corolla is 2.5-4.5cm in length and the throat is partially open (Stace 2010). This is a taxon of variable pubescence, but is usually hairy in the upper parts of the plant (Stace 2010) including the inflorescences which present simple hairs in the base of the calyx (MVM pers. obs.). Of garden origin *Mimulus × robertsii* can occasionally arise in the wild; this hybrid is produced by crosses of *Mimulus guttatus* with *Mimulus luteus* var. *rivularis, M. luteus* var. *variegatus* or *Mimulus × smithii* Paxton (the latter a hybrid between *Mimulus luteus* var. *rivularis* and *Mimulus luteus* var. *variegatus*, which is phenotypically very similar to *Mimulus luteus* var. *youngana*) (Stace 2010). In the UK it can be found up to 610 m (Ochil Hills, Scotland), and is suggested to be the commonest taxon of high ground (Preston et al. 2002, Stace 2010).

*Mimulus peregrinus* resembles *Mimulus × robertsii* rather closely in habit, size and general vegetative and floral morphology, suggesting a close affinity between these two taxa (Table 1). *Mimulus × robertsii* and *Mimulus peregrinus* can be distinguished by their differences in chromosome number, pollen and seed fertility, pollen grain size, and stomata size (Table 1). *Mimulus peregrinus* presents consistently high levels of pollen fertility (0.86 ± 0.04) and is capable of producing abundant seed set following artificial pollination. In contrast, both natural and artificial specimens of *Mimulus × robertsii* present very low levels of pollen viability (proportion of viable pollen = 0.05 ± 0.01, for both naturalized (N *=* 7) and synthetic hybrids (N = 15)), and do not set seed following artificial pollination (Roberts 1964) (see also Table 1). In addition, the two taxa differ markedly in chromosome number: *Mimulus × robertsii* is a triploid (e.g. 2n = 45), while *Mimulus peregrinus* has twice as many chromosomes (2n = 92), and this difference in genome size is clearly seen in flow cytometry analysis of DAPI-stained nuclei (Fig. 3). Finally, associated with the different genome size of the two taxa, *Mimulus peregrinus* has larger pollen grains, larger seeds, and larger stomata than *Mimulus robertsii* (Table 1).

(5) Other hybrids. *Mimulus × burnetii* S. Arn. (*Mimulus guttatus* × *Mimulus cupreus*) is a sterile triploid (2n = 45) with copper-coloured corolla, and often presenting a petaloid calyx (Stace 2010). *Mimulus × polymaculus* Silverside nom. nud. (*Mimulus guttatus* × (*Mimulus luteus* × *Mimulus cupreus*)) is also a sterile triploid that differs from *Mimulus × burnetii* in having dark blotches in the corolla lobes. Both can be easily distinguished from *Mimulus peregrinus* based on corolla colour, calyx morphology, fertility, and chromosome number. *Mimulus × maculosus* W. Bull ex T. Moore (*Mimulus cupreus* × *Mimulus luteus*) and *Mimulus × hybridus* Siebert & Voss (*Mimulus cupreus* × *Mimulus × smithii*) are fertile hybrids with variably-coloured corollas, often copper-coloured or with blotches on the petal lobes. They can both be easily distinguished from *Mimulus peregrinus* by their corolla colours, lack of abundant simple hairs in the keels of the calyx, and evenly triangular, flat teeth in the leaf margins. Chromosome numbers for these latter two hybrids are not yet available, but it is to be expected that they are similar to their parental species (2n = 60-62).

### Putative origin and distribution of *Mimulus peregrinus*

The intermediate floral and vegetative characteristics of *Mimulus peregrinus* between *Mimulus guttatus* and *Mimulus luteus*, as well as its close morphological similarity to *Mimulus × robertsii* clearly suggest a hybrid origin for this new taxon associated with a polyploidization event. The alternative, that *Mimulus peregrinus* is an autopolyploid derivative of a pure *Mimulus guttatus* or *Mimulus luteus* seems highly unlikely based on vegetative and floral characteristics of the different taxa (Table 1). Moreover, both chromosome counts and genome size data are inconsistent with the expectations of an early generation autopolyploid of either *Mimulus guttatus* or *Mimulus luteus* or a backcross between *Mimulus × robertsii* and either parent (Fig. 3). The fact that *Mimulus peregrinus* presents approximately twice the number of chromosomes and has double the amount of DAPI-staining DNA than a common cytotype of *Mimulus × robertsii* (Fig. 3), immediately suggests that the most parsimonious explanation for the origin of *Mimulus peregrinus* is through hybridization between *Mimulus guttatus* and *Mimulus luteus* linked to a polyploidization event. Given that *Mimulus peregrinus* was indentified amongst a large population of *Mimulus × robertsii*, a possible origin of this new taxon is via genome doubling of the triploid hybrid.

The known distribution of *Mimulus peregrinus* iscurrently restricted to a single locality in Scotland. A preliminary examination of herbarium specimens at the Royal Botanic Gardens in Edinburgh did not uncover any hybrid specimens that were obviously fertile. However, the widespread distribution of *Mimulus × robertsii* in the UK suggests, along with anecdotal records of fertility in hybrids (Silverside 1998), may suggest that *Mimulus peregrinus* could be significantly under recorded, and future studies are required to determine its actual distribution.

It is well known that polyploidization can act as a mechanism restoring fertility even in highly sterile triploid hybrids (Dobzhansky 1937, Stebbins 1950, Grant 1971, Ramsey and Schemske 1998, Briggs and Walter 2000), and polyploidization has resulted in the evolution of other non-native allohexaploid species from highly sterile triploids in the UK (e.g. *Senecio cambrensis*, 2n = 6x; Abbott and Lowe 2004). While firmly establishing the origin and distribution of *Mimulus peregrinus* must await further ecological and genetic work, the discovery of this taxon provides an exciting opportunity to study the recent evolution of a new allopolyploid British species.

## Supplementary Material

XML Treatment for Mimulus peregrinus
